# The Ecto-5′nucleotidase/CD73 Mediates *Leishmania amazonensis* Survival in Macrophages

**DOI:** 10.1155/2022/9928362

**Published:** 2022-02-11

**Authors:** Bijay Bajracharya, Deena Shrestha, André Talvani, Ricardo Gonçalves, Luís Carlos Crocco Afonso

**Affiliations:** ^1^Laboratory of Immunoparasitology, Biological Sciences Department, ICEB-Federal University of Ouro Preto, Minas Gerais, Brazil; ^2^Laboratory of Immunobiology of Inflammation, Biological Sciences Department, ICEB-Federal University of Ouro Preto, Ouro Preto, Minas Gerais, Brazil; ^3^General Pathology Department, ICB, Federal University of Minas Gerais, Belo Horizonte, Minas Gerais, Brazil

## Abstract

Endogenous nucleotides produced by various group of cells under inflammatory conditions act as potential danger signals *in vivo*. Extracellularly released nucleotides such as ATP are rapidly hydrolyzed to adenosine by the coordinated ectonucleotidase activities of CD39 and CD73. *Leishmania* is an obligate intracellular parasite of macrophages and capable of modulating host immune response in order to survive and multiply within host cells. In this study, the activity of CD73 induced by *Leishmania amazonensis* in infected macrophages has been investigated and correlated with parasite survival and infection *in vitro*. For this, the expression of CD39 and CD73, by flow cytometry, in murine peritoneal macrophages infected with metacyclic promastigotes of *L. amazonensis* has been analyzed. Our results showed that *L. amazonensis-*infected macrophages, unlike LPS-treated macrophages, increased CD73 expression. It was also noted that when CD73 enzymatic activity was blocked by *α*, *β*-methyleneadenosine 5′-diphosphate sodium salt (APCP), macrophage parasitism was significantly decreased. Interestingly, these effects were not associated with the production of TNF-*α*, IL-10, or nitric oxide (NO). Together, these data demonstrate that *L. amazonensis* induces a regulatory phenotype in macrophages, which by activating the CD39/CD73 pathway allows parasite survival through the action of immunomodulatory adenosine receptors.

## 1. Introduction

*Leishmania* are intracellular parasites that live and multiply within macrophages in the mammalian host. In order to survive in these cells, *Leishmania* amastigotes must resist or inhibit their microbicidal mechanisms [1]. Cutaneous leishmaniasis associated with *Leishmania amazonensis* infection is severe in both humans and experimental models [2, 3]. It is characterized by uncontrolled parasite replication and profound host immunosuppression [4–7]. This parasite has been shown to alter the host cell defense mechanisms in several ways such as inhibition of antigen presentation and inhibition of reactive oxygen species (ROS) and nitric oxide (NO) production [8–10]. The underlying mechanisms involved in the manipulation of the macrophage activation, however, remain largely unclear.

Accumulating evidence supports that extracellular ATP released at the sites of infection and its degradation product, adenosine, function as potent immune modulators that mediate both pro- and anti-inflammatory pathways, depending on the agonist concentration and receptor subtypes expressed by the cell [11]. The increase in extracellular ATP concentration leads to the activation of NLRP3 inflammasome and subsequent release of IL-1*β* and TNF-*α* by macrophages [12, 13]. Extracellular adenosine formation generally results from the sequential hydrolysis of extracellular ATP by the combined action of an ectonucleoside triphosphate diphosphohydrolase (CD39) followed by ecto-5′ nucleotidases (CD73) [14]. Adenosine, by acting on P1 receptors, modulates the activation of macrophages, reducing inflammatory and increasing regulatory cytokine production [15]. The ATP/adenosine ratio is determined by the activity of the CD39/CD73 pathway, which can be altered by pathophysiological events and ultimately defines the outcome of infections, inflammation, and injuries [16, 17].

The capacity of a pathogen to generate extracellular adenosine through the expression of ectonucleotidases has been identified as an important virulence factor [18]. Our laboratory has been successful to demonstrate that the level of ectonucleotidase activity in promastigote forms of *Leishmania* is associated with the severity of disease in the murine experimental model and may be involved in the outcome of distinct clinical manifestations in patients [19–22]. In the case of *Leishmania*, however, once the parasite is internalized, its ectonucleotidase activity should cease to influence the levels of extracellular ATP and adenosine near the host cell [21]. Interestingly, however, we have also demonstrated that upon infection of dendritic cells, *Leishmania* promastigotes induce the upregulation of CD39 and CD73 on infected dendritic cells, thus increasing the ability of these cells to produce extracellular adenosine [23]. This pathway in turn has further shown to impair dendritic cell activation through immunomodulating A2b receptors and triggering cAMP pathways in infected cells [24].

Based on all our previous data [19–24], we hypothesized that CD39 and CD73 enzymes may also influence macrophage-*Leishmania* interaction through purinergic receptors, thereby affecting the parasite survival and multiplication within macrophages. *Leishmania* parasites in *Leishmania*-infected macrophages may utilize these molecules during their early host macrophage interaction downregulating host immune response leading to uncontrolled parasite multiplication and macrophages inactivation. It has been demonstrated by Cohen et al. [15] that macrophages self-regulate their activation status by means of adenosine production mediated by CD39 and activation of A2b adenosine receptor, a process that endows the macrophage with regulatory capacity [15]. In brief, our study will try to address CD39/CD73 pathway as one of the possible regulatory mechanisms induced by *L. amazonensis* in macrophages making them hostile for parasite development and proliferation.

## 2. Material and Methods

### 2.1. Animals

The C57BL/6 mice (8-12 weeks, both male and female) were used for our study. Mice were housed and maintained at the central animal facility in the Universidade Federal de Ouro Preto (UFOP). All animal experiments and procedures were approved by the institution's committee on ethical handling of laboratory animals (Protocol 2012/56).

### 2.2. Preparation of Parasites

*L. amazonensis* (IFLA/BR/1967/PH8) promastigotes were grown at 25°C in Grace's medium (Sigma-Aldrich Inc., St. Louis, MO, USA) supplemented with 10% inactivated fetal bovine serum (FCS-LGC Biotecnologia, Cotia, SP, Brazil), 2 mM l-glutamine (GIBCO BRL-Life Technologies, Grand Island, NY, MO, EUA), and 100 U/ml penicillin G (USB Corporation, Cleveland, OH, USA), pH 6.5. Five-day-old stationary phase promastigotes were used for metacyclic isolation and purification [19, 25].

### 2.3. Carboxyfluorescein Succinimidyl Ester (CFSE) Labeling

Purified metacyclics (6 × 10^7^ parasites/ml). The carboxyfluorescein diacetate succinimidyl ester (CFSE) dye was laid over the 50 *μ*l of PBS and incubated with parasites (final concentration -5 *μ*M) at 37°C for 10 min in the dark [26]. Parasites were then washed with PBS/10% FBS, pH 7.2, and then suspended in Dulbecco's modified eagle's medium (DMEM-Sigma-Aldrich, Missouri, EUA) with 10% FBS, 2 mM l-glutamine, 100 U/ml penicillin G, 25 mM N-2-hydroxiethylpiperazine-N9-2-ethanosulfonic acid (HEPES; USBiological, Swampscott, MA, USA), 1.2 mM sodium bicarbonate (Vetec Quimica Fina, RJ, Brazil), and 50 *μ*M 2-mercaptoethanol (Pharmacia Biotech AB, Uppsala, Sweden) pH 7.2 prior to addition to macrophage cultures. Later, CFSE-tagged parasites were measured in FACS diagram, and the fluorescence was captured in channel 1 in BD FACSCalibur.

### 2.4. Resident Peritoneal Macrophages

Mice were euthanized, and the abdomen was gently massaged, and peritoneal lavage was collected after injection of 10 ml ice-cold PBS with 16G needle [27]. Cells were centrifuged at a speed of 210×*g*, 4°C for 10 min, and were resuspended in supplemented DMEM. Cell viability was confirmed by trypan blue exclusion (Sigma-Aldrich). For the preparation of rested resident macrophages, naïve macrophages were incubated at 37°C/5%CO_2_ for 24 to 72 h in supplemented DMEM. Later, macrophages were detached using 0.05% EDTA in PBS followed by washing with PBS and incubation with trypsin for 10-15 min. The expression of CD39 and CD73 molecules in freshly harvested macrophages were studied over period of 24 h, 48 h, and 72 h *in vitro*.

For macrophage CD39 and CD73 expression *in vitro* studies, 72 h rested resident macrophages (5 × 10^5^) were infected with metacyclics forms (3 parasites/cell) of CFSE-labeled *L. amazonensis* in supplemented DMEM. In certain groups, rested macrophages were treated with lipopolysaccharide (LPS) obtained from *E. coli* (Sigma-Aldrich) at the concentration of 5 *μ*g/ml. Cells were then incubated at 33°C/5%CO_2_ for 24 h or 48 h.

In *in vitro* infection studies, resident peritoneal cells (5 × 10^5^) were seeded in 24-well plates provided in each well with grease-free sterile coverslips of 13 mm diameter for 72 h. Any unbound resident cells were removed by washing two times with Phosphate buffer saline (PBS) before infection. Fresh medium was added to the rested macrophages which were then infected with metacyclic forms of *L. amazonensis* in a ratio of 3 parasites/cell. Cells were incubated at 33°C/5%CO_2_ for 3 h, and excess parasites were then removed by washing twice with PBS. Cells were further incubated at 33°C/5%CO_2_ for 24 h or 48 h.

For CD73 inhibition experiments, *α*, *β*-methyleneadenosine 5′-diphosphate sodium salt (APCP) (Sigma-Aldrich) was added at a concentration of 200 *μ*M after 3 h of infection and was kept throughout the infection. The inhibitor was dissolved in PBS.

In all conditions, coverslips were removed 3 h, 24 h, and 48 h post infection from macrophage culture plates. Coverslips were then fixed in methanol for 10 min (Vetec Fine Chemistry), dried, and stained using Panótico Rápido kit (Renylab química e farmacêutica, MG, Brazil) following manufacturer's instructions. Coverslips were analyzed using an Olympus BX50 optical microscope (Olympus, Center Valley, PA, USA). A minimum of 200 macrophages per coverslip was examined, and the number of uninfected, infected, and amastigotes in infected macrophages was recorded.

### 2.5. Flow Cytometry

All samples were washed twice with PBS (210×*g*, 4°C, 10 min). Cells were then resuspended in 0.2% bovine serum albumin (0.2% BSA/PBS) and Fc-blocked (purified rat anti-mouse CD16/CD32, clone 2.4G2, BD Pharmingen) for 15 min in ice. The cells were then washed and stained with anti-mouse F4/80PE-CY7 antibody (clone BM8, BioLegend), anti-mouse CD73PE antibody (clone Ty/11.8, eBiosciences), and anti-mouse CD39Alexa Fluor 647 (clone 24DMS1, eBiosciences) antibodies in ice for 30 min. The cells were washed with PBS and then fixed in 250 *μ*l fixation solution (1% paraformaldehyde, 47.7 mM sodium cacodylate, and 113 mM NaCl; pH 7.2). Samples were analyzed using a BD FACSCaliburTM flow cytometer. All cytometric analyses were performed by using Flow Jo version 7.6.5 (Tree Star, Ashland, OR, USA).

### 2.6. Cytokine and Nitric Oxide Measurements

TNF-*α* and IL-10 in cell culture supernatants were determined by ELISA kits (Mouse TNF-*α* DuoSet catalogue DY410, Mouse IL-10 Duoset catalogue DY417E from R&D system). Assays were performed according to the manufacturer's instructions. Nitric oxide in cell culture supernatants was measured by spectrophotometric assay based on the Griess reaction [28].

### 2.7. Statistical Analysis

Data were expressed as mean ± SD. Several group data were analyzed by one-way analysis of variance (ANOVA) followed by Bonferroni posttest or by Newman-Keuls multiple comparison test. Two group comparisons were performed by paired Student's test. *p* value <0.05 was considered statistically significant.

## 3. Results

### 3.1. Peritoneal Resident Macrophages Express Both CD39 and CD73 In Vivo

**Figure 1:**
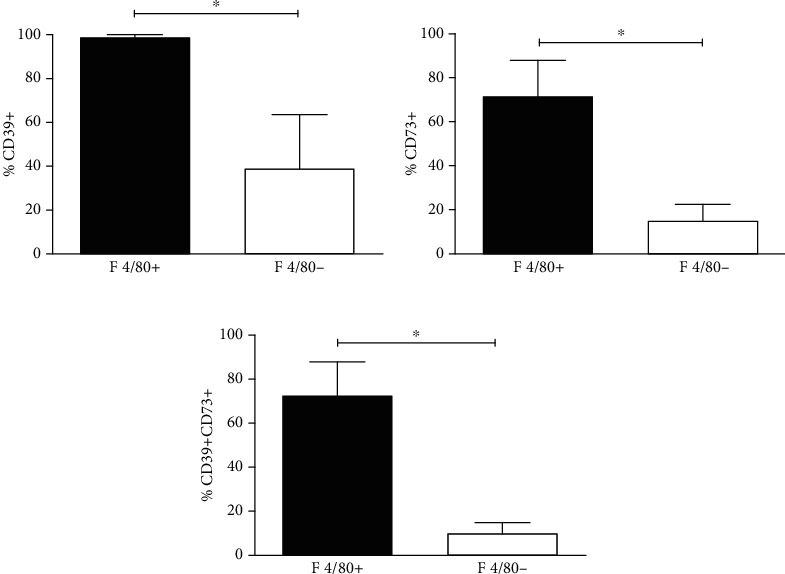
Expression of CD39 and CD73 in naïve macrophages in *in vivo*. Resident macrophages were harvested from the peritoneum of naïve C57BL/6 mice using ice-cold 10 ml PBS. After centrifugation and resuspension in 0.2% bovine serum albumin (0.2% BSA/PBS) and Fc-blocked (purified rat anti-mouse CD16/CD32, clone 2.4G2, BD Pharmingen), the harvested cells were labeled with anti-murine F4/80, anti-CD39, and anti-CD73 antibodies and then were analyzed by flow cytometry. Indicated in the figure, the percentage of cells expressing (a) CD39, (b) CD73, and (c) CD39CD73 in F4/80^+^ and F4/80^−^ cells from total peritoneal population. This result is the mean ± SD of at least 3 independent experiments. ^∗^*p* < 0.05 means the statistical difference in the expression of CD39 and CD73 between F4/80^+^ and F4/80^−^ cell populations using paired two-tailed Student's *t*-test.

Our first attempt was to investigate whether resident peritoneal macrophages, which represent a significant proportion of the total peritoneal population, express ectonucleotidases on their surfaces. In freshly harvested total peritoneal resident population, we observed two sets of distinct cell population when marked with F4/80^+^ antibody. Within the F4/80^+^ population (macrophages), we observed that all cells were CD39+, while only 39% of F4/80^−^ (other than macrophage cell population) expressed this molecule (Figure 1(a)). Interestingly, only 72% of F4/80^+^ cells expressed CD73 markers (Figure 1(b)). In combination, macrophages represented majority of cells expressing both ectonucleotidases (Figure 1(c)).

### 3.2. Resident Macrophages Downregulate CD73 Expression In Vitro

**Figure 2:**
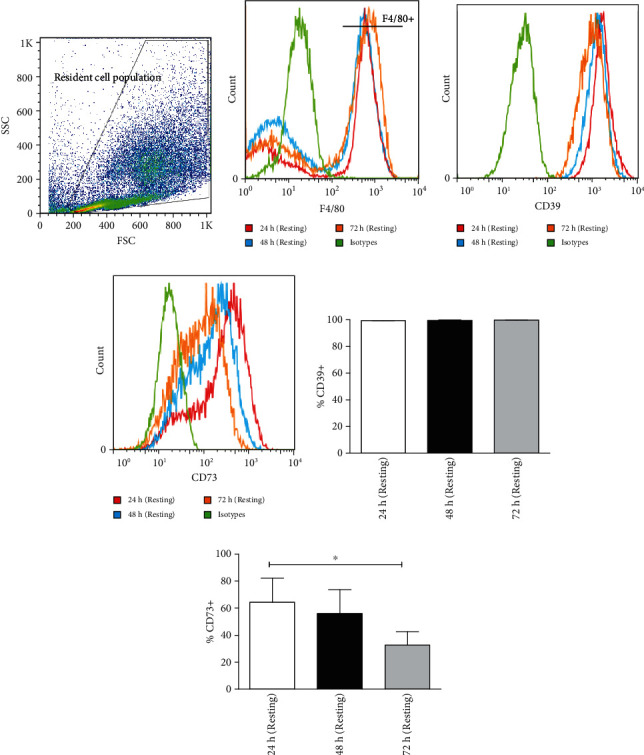
Resident macrophages down regulate CD73 expression in *in vitro*. Resident macrophages were harvested from naïve C57BL/6 mice. Total peritoneal cell population was counted, and viability of the cells was determined by trypan blue. 5 × 10^5^ cells were cultured *in vitro* at 37°C and was left for 24 h, 48 h, and 72 h of resting and subsequently incubated for 24 h at 33°C/5%CO_2_ before analysis by flow cytometry (a). Size and granularity for total peritoneal population (b). F4/80^+^ cells were first gated from total peritoneal population and then cells expressing CD39 and CD73 in F4/80^+^ population from 24 h, 48 h, and 72 h rested macrophages were overlaid in the histograms (c and d). The percentage of cells expressing (e) CD39 and (f) CD73 is represented in bar diagrams for macrophages. This result is representative of 3 independent experiments. ^∗^*p* < 0.05 indicates the statistical difference using paired two-tailed Student's *t*-test.

We found that CD39 and CD73 enzymes were abundantly present in macrophages ex vivo, but the characteristics of these molecules in *in vitro* were yet to be elucidated. The CD39 and CD73 expressions in resident macrophages were, therefore, characterized at different time points after harvest from the peritoneum. For this, cells were incubated for 24, 48, and 72 h in the absence of any external stimulus. As shown in Figure 2, although the percentage of macrophages remained constant during the incubation period (Figure 2(b)), macrophages spontaneously downregulated CD73 without altering their CD39 expression *in vitro* (Figures 2(c)–2(f)). The level of the CD73 expression gradually decreased over the incubation period. This result suggests that culture conditions may have an important role in the expression of CD73 *in vitro*. Alternatively, the expression of CD73 could have been activated by the harvesting procedure and then gradually returned to a steady state condition.

### 3.3. L. amazonensis Increases CD73 Expression in Rested Macrophages but Does Not Affect Cytokine and NO Production

**Figure 3:**
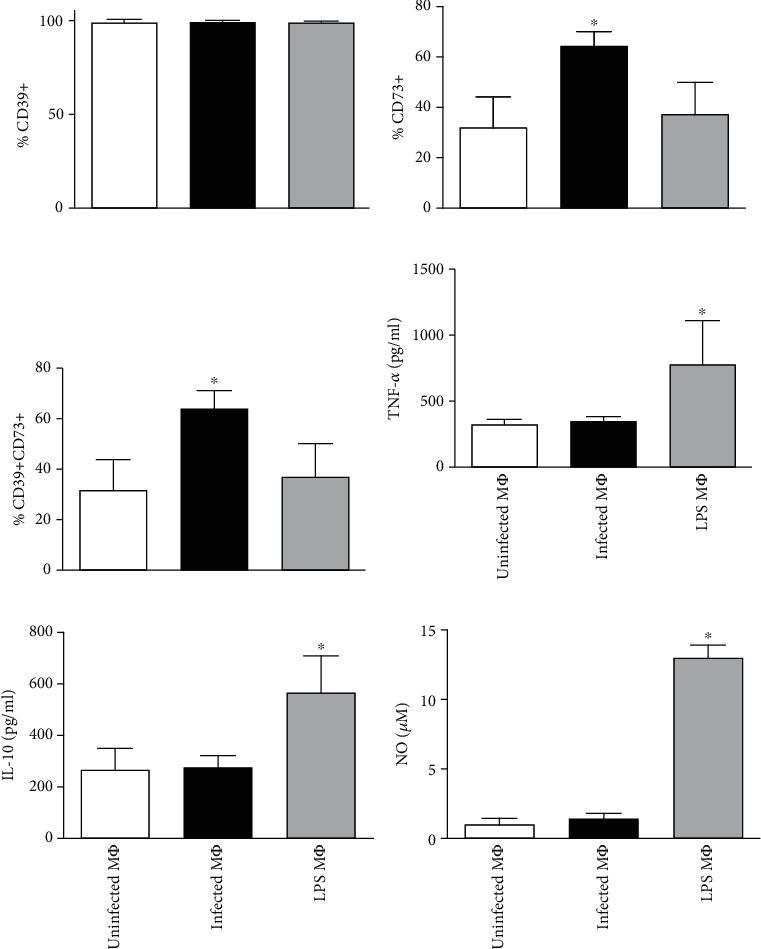
*Leishmania amazonensis* increases CD73 expression but does not alter cytokines and nitric oxide (NO) production. Resident cell population was collected from naïve C57BL/6 mice and rested for 72 h prior to infection as previously discussed in the methodology. The cells were infected with CFSE-tagged metacyclics of *L. amazonensis*, and additionally, another group was treated with 5 *μ*g/ml of LPS. The cells were further incubated for 24 h at 33°C/5%CO_2_. Supernatant from all groups was collected for the measurement of TNF-*α*, IL-10, and NO. Macrophages (MФ) expressing (a) CD39, (b) CD73, (c) CD39CD73, and immunoassays from culture supernatants for (d) TNF-*α*, (e) IL-10, and (f) nitric oxide production in treated macrophages are shown in bar diagrams. This result is the mean ± SD of at least 3 independent experiments. ^∗^*p* < 0.05 indicates the statistical significance between infected and control groups using one-way analysis of variance (ANOVA) followed by Bonferroni posttest.

Having shown that the rested resident macrophages decrease CD73 expression upon incubation, our next objective was to observe if the infection by *L. amazonensis* induces any change in CD39 and CD73 surface expressions in macrophages. We exposed 72 h rested macrophages to CFSE-labeled metacyclics of *L. amazonensis*. Interestingly, it was found that although the percentage of CD39^+^ cells did not alter after infection (Figure 3(a)), a significant increase in the CD73 expression was observed in this group (Figure 3(a)). LPS treatment did not affect either CD39 or CD73 expression, suggesting that LPS-activated macrophages do not upregulate CD73.  Figure 3(c) demonstrates that the combined expression of CD39 and CD73 is higher amongst *L. amazonensis*-infected macrophages when compared to unstimulated or LPS-treated cells. Furthermore, when the infection was prolonged to 48 h of incubation after 72 h rested period, we found that the infected macrophages still kept CD73 expression higher than the control groups (data not shown).

In addition to the expression of ectonucleotidases, TNF-*α*, IL-10, and NO production by treated macrophages was evaluated. As shown in Figures 3(d)–3(f), rested macrophages responded to LPS treatment by producing TNF-*α*, IL-10, and NO. On the other hand, *L. amazonensis*-infected macrophages did not increase the production of these cytokines neither NO indicating that these cells were fully capable to react to inflammatory stimuli but not to the parasite.

Together, these data indicate that *L. amazonensis* not only inhibits cytokine and NO production but also upregulates CD73 by rested macrophages upon infection. The increased capacity of infected cells to hydrolyze extracellular ATP and produce adenosine, associated to the lack of activation to produce NO, may have negative effects on the ability of the cell to control parasite multiplication.

### 3.4. CD73 Activity Allows for Parasite Survival in Infected Macrophages

**Figure 4:**
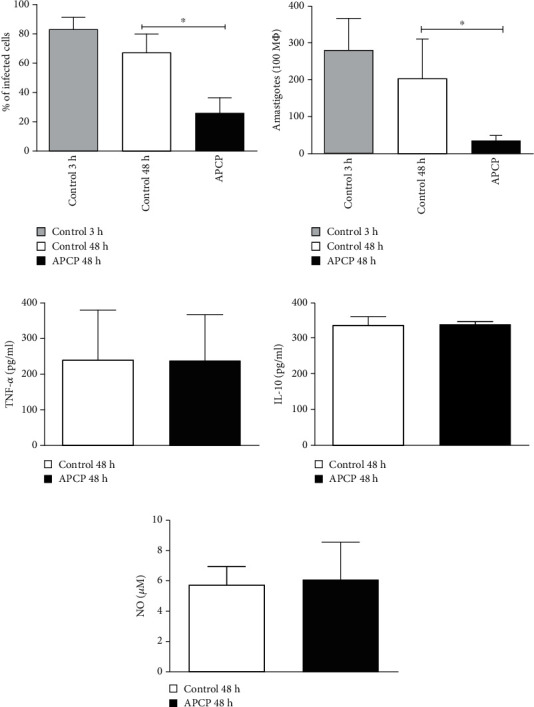
CD73 activity determines survival of *L. amazonensis*. Resident macrophages were obtained from naïve mice by injecting 10 ml of ice-cold PBS into the peritoneal cavity and rested for 72 h at 37°C. The cells were then infected with the metacyclic forms of the parasites in a ratio of 1 : 3 and allowed for the parasites to interact for 3 h at 33°C/5%CO_2_. Extracellular parasites were washed away, and the inhibitor *α*, *β*-methyleneadenosine 5′-diphosphate sodium salt (APCP) was added against CD73 at a concentration of 200 *μ*M. This treatment was left throughout the period of infection. Supernatant was collected from these groups after 48 h. The percentage of infection (a) and the amastigote number per 100 macrophages (b) were shown compared with 3 h and 48 h of incubation with or without inhibitor. Production of cytokines, (c) TNF-*α*, (d) IL-10, and (e) nitric oxide (NO) in treated groups is also illustrated here. Data are the mean ± SD from 3 independent experiments. ^∗^*p* < 0.05 is the statistical difference between control and treated groups using repeated measures of ANOVA followed by Newman-Keuls multiple comparison test.

Once upregulation of CD73 on the membrane of *L. amazonensis*-infected macrophages was demonstrated, we sought to determine whether the activity of these enzymes is crucial for the survival of the parasites. For this, resident macrophages were seeded and were rested for 72 h prior to infection in the presence of inhibitor of CD73. Since the use of inhibitors at the time of infection could interfere with similar enzymes present on the surface of these parasites [20], APCP, inhibitor for CD73, was added after the parasites had been incubated with the macrophages for 3 h and subsequently removed by washing. There was no death or changing in cellular viability concerning the incubation with *β*-methyleneadenosine 5′-diphosphate sodium salt inhibitor. Our data showed that already after 24 h of infection, treatment of infected macrophages with APCP reduced parasitism (data not shown) and by 48 h of incubation with this inhibitor, the percentage of infected macrophages and the number of amastigotes per 100 macrophages were significantly decreased (Figures 4(a) and 4(b)). Interestingly, the reduction in parasite survival in macrophages that had been treated with ectonucleotidase inhibitors was not associated with alterations in the levels of TNF-*α*, IL-10, or NO production as shown in Figures 4(c)–4(e), suggesting that survival of *L. amazonensis* within infected macrophages is dependent on the activity of CD73 surface enzymes, rather than cytokine-mediated NO production.

Our results demonstrate that *L. amazonensis* upregulates CD73 in resident macrophages and its survival within the cell is strictly dependent on CD73 enzymatic activity.

## 4. Discussion

Macrophages and *Leishmani*a have a complex relationship. In the presence of an adequate immune response, macrophages can be activated and kill intracellular amastigote forms of *Leishmania* [29]. However, as in the case of *L. amazonensis* infection, even in the presence of a Th1 response, capable of eliminating other *Leishmania* species, the parasite survives within the infected macrophages, indicating its ability to control macrophages activation [4, 6, 7]. Several studies have pointed out that CD39 and CD73, which are present in many immune cells, play an important role in the infections [30–33], inflammation [34], and immune modulation [16, 35–38].

Interestingly, it was observed that all freshly harvested resident peritoneal macrophages (F4/80^+^) expressed CD39 which is in agreement with the previous studies showing that CD39 is one of the predominant markers for mature macrophages [39]. The CD73 expression, on the other hand, was limited only to 72% of the F4/80^+^ population which suggests that the expression of this enzyme is dependent on other factors such as the activation state of the cell or that it is restricted to a certain subpopulation of macrophages. Remarkably, it was observed that the expression CD73 in resident macrophages decreased significantly with incubation in culture medium for 72 hr. The expression of CD73 in resident peritoneal macrophages has been described in the previous studies [40, 41]; however, to the best of our knowledge, no previous study has evaluated the expression of this enzyme after an extended period of incubation such as the one used in the present study.

During infection, macrophages secrete ATP via pannexin channels [42–44] or P2X7 receptors [45]. Accumulation of extracellular ATP in the surrounding environment may induce excessive inflammatory reactions at the site of infection, and in addition, it may also induce apoptosis of the cells that produce them [46–48]. Cohen et al. [15] showed that in the absence of CD39 activity, the accumulation of extracellular ATP secreted by TLR stimulated macrophages leads to an exacerbated inflammatory response that can be harmful for the host. Our results showed that while CD39 expression in *L. amazonensis*-infected macrophages was not altered with the parasite, CD73 was significantly upregulated. The fact that infection by *L. amazonensis* increases CD73 expression, our result suggests that the infected macrophages would present a higher regulatory capacity than that of LPS-treated macrophages. The increase in the CD73 expression by infected macrophages corroborates the previous findings from our laboratory which demonstrated that the CD39 and CD73 expressions are increased in infected dendritic cells [23] indicating that the upregulation of ectonucleotidase expression is a conserved mechanism of inhibiting the establishment of immune response by the parasite on various cell types.

The importance of adenosine production by the infected macrophage in the regulation of cellular activation was established by the decreased parasite survival in cells treated with CD73 inhibitor (Figure 4). Macrophages treated with APCP were capable of, at least partially, control parasite survival within the infected cell. Similar findings were also observed in *L. donovani*-infected macrophages when enzymes CD39 and CD73 were blocked by the inhibitors at concentrations similar to those used in the present study [44]. The same study shows that the enzyme activity (release of Pi by ATP and AMP degradation) in the presence of an inhibitor decreased significantly when CD39 and CD73 enzyme activities were inhibited [44]. All this evidence supports the hypothesis that parasite survival is dependent on the enzyme activity of CD39 and CD73.

The end result of the combined activity of CD39 and CD73 is the production of extracellular adenosine which, by acting on the A2a or A2b receptors, will downmodulate macrophage microbicidal mechanisms such as NO and ROS production. We did not address in the present study which adenosine receptor was involved in the downmodulation of the microbicidal activity and cytokine production of infected macrophages. However, the involvement of A2a and A2b receptors in *L. donovani* infection in macrophages was highlighted in a recent study [44]. Furthermore, in the same study, it was demonstrated that if these receptors were blocked by specific antagonists, parasite survival was moderately decreased [44]. These results implicate that adenosine production rather than decrease in the levels of ATP, which could activate the macrophage if its hydrolysis was impaired, is the important step in parasite growth restriction. Moreover, previous findings [49] from our laboratory in J774 cells infected with *L. amazonensis* supported the evidence that when A2b receptors were blocked by MRS1754, parasite survival within macrophages decreased significantly.

In summary, our results demonstrated that upon infection by *L. amazonensis*, macrophages differentiate into regulatory cells and were capable of hydrolyzing extracellular ATP and produce adenosine. By activation of the CD39CD73 enzymes, mainly of the CD73, the inhibition of adenosine production/activity inhibits parasite survival within the infected macrophage. In perspective, defining the role of adenosine production on the control of the host immune response may present new alternatives for the control of Leishmania and/or other trypanosomatids.

## Data Availability

The data of this manuscript will be made available by the corresponding author on a reasonable request.

## References

[1] Olivier M., Gregory D. J., Forget G. (2005). Subversion mechanisms by which Leishmania parasites can escape the host immune response: a signaling point of view. *Clinical Microbiology Reviews*.

[2] Scorza B. M., Carvalho E. M., Wilson M. E. (2017). Cutaneous manifestations of human and murine leishmaniasis. *International Journal of Molecular Sciences*.

[3] de Oliveira Cardoso F., da Silva Freitas de Souza C., Gonçalves Mendes V., Abreu-Silva A. L., da Costa S. C. G., da Silva Calabrese K. (2010). Immunopathological studies ofLeishmania amazonensisInfection in resistant and in susceptible mice. *The Journal of Infectious Diseases*.

[4] Afonso L. C., Scott P. (1993). Immune responses associated with susceptibility of C57BL/10 mice to Leishmania amazonensis. *Infection and Immunity*.

[5] Scott P., Novais F. O. (2016). Cutaneous leishmaniasis: immune responses in protection and pathogenesis. *Nature Reviews. Immunology*.

[6] Ji J., Sun J., Soong L. (2003). Impaired expression of inflammatory cytokines and chemokines at early stages of infection with Leishmania amazonensis. *Infection and Immunity*.

[7] Christensen S. M., Belew A. T., El-Sayed N. M., Tafuri W. L., Silveira F. T., Mosser D. M. (2019). Host and parasite responses in human diffuse cutaneous leishmaniasis caused by L. amazonensis. *PLoS Neglected Tropical Diseases*.

[8] Martínez-López M., Soto M., Iborra S., Sancho D. (2018). Leishmania hijacks myeloid cells for immune escape. *Frontiers in Microbiology*.

[9] Horta M. F., Mendes B. P., Roma E. H. (2012). Reactive oxygen species and nitric oxide in cutaneous leishmaniasis. *Journal of Parasitology Research*.

[10] Meier C. L., Svensson M., Kaye P. M. (2003). Leishmania-induced inhibition of macrophage antigen presentation analyzed at the single-cell level. *Journal of Immunology*.

[11] Ralevic V., Burnstock G. (1998). Receptors for purines and pyrimidines. *Pharmacological Reviews*.

[12] Riteau N., Baron L., Villeret B. (2012). ATP release and purinergic signaling: a common pathway for particle-mediated inflammasome activation. *Cell Death & Disease*.

[13] Hide I., Tanaka M., Inoue A. (2000). Extracellular ATP triggers tumor necrosis factor-*α* release from rat microglia. *Journal of Neurochemistry*.

[14] Yegutkin G. G. (2008). Nucleotide- and nucleoside-converting ectoenzymes: important modulators of purinergic signalling cascade. *Biochimica et Biophysica Acta*.

[15] Cohen H. B., Briggs K. T., Marino J. P., Ravid K., Robson S. C., Mosser D. M. (2013). TLR stimulation initiates a CD39-based autoregulatory mechanism that limits macrophage inflammatory responses. *Blood*.

[16] Antonioli L., Pacher P., Vizi E. S., Hasko G. (2013). CD39 and CD73 in immunity and inflammation. *Trends in Molecular Medicine*.

[17] Dwyer K. M., Deaglio S., Gao W., Friedman D., Strom T. B., Robson S. C. (2007). CD39 and control of cellular immune responses. *Purinergic Signal*.

[18] Sansom F. M., Robson S. C., Hartland E. L. (2008). Possible effects of microbial ecto-nucleoside triphosphate diphosphohydrolases on host-pathogen interactions. *Microbiology and Molecular Biology Reviews*.

[19] de Almeida Marques-da-Silva J. C., de Oliveira A. B., Figueiredo J. D. (2008). Extracellular nucleotide metabolism in _Leishmania_ : influence of adenosine in the establishment of infection. *Microbes and Infection*.

[20] de Souza M. C., de Assis E. A., Gomes R. S. (2010). The influence of ecto-nucleotidases on _Leishmania amazonensis_ infection and immune response in C57B/6 mice. *Acta Tropica*.

[21] Leite P. M., Gomes R. S., Figueiredo A. B. (2012). Ecto-nucleotidase activities of promastigotes from Leishmania (Viannia) braziliensis relates to parasite infectivity and disease clinical outcome. *PLoS Neglected Tropical Diseases*.

[22] Maioli T. U., Takane E., Arantes R. M., Fietto J. L., Afonso L. C. (2004). Immune response induced by New World Leishmania species in C57BL/6 mice. *Parasitology Research*.

[23] Figueiredo A. B., Serafim T. D., Marques-da-Silva E. A., Meyer-Fernandes J. R., Afonso L. C. (2012). Leishmania amazonensis impairs DC function by inhibiting CD40 expression via A2B adenosine receptor activation. *European Journal of Immunology*.

[24] Figueiredo A. B., Souza-Testasicca M. C., Mineo T. W. P., Afonso L. C. C. (2017). Leishmania amazonensis-induced cAMP triggered by adenosine A_2B_ receptor is important to inhibit dendritic cell activation and evade immune response in infected mice. *Frontiers in Immunology*.

[25] Spath G. F., Beverley S. M. (2001). A Lipophosphoglycan-Independent Method for Isolation of Infective _Leishmania_ Metacyclic Promastigotes by Density Gradient Centrifugation. *Parasitology*.

[26] Goncalves R., Vieira E. R., Melo M. N., Gollob K. J., Mosser D. M., Tafuri W. L. (2005). A sensitive flow cytometric methodology for studying the binding of L. chagasi to canine peritoneal macrophages. *BMC Infectious Diseases*.

[27] Zhang X., Goncalves R., Mosser D. M. (2008). The isolation and characterization of murine macrophages. *Current Protocols in Immunology*.

[28] Green L. C., Wagner D. A., Glogowski J., Skipper P. L., Wishnok J. S., Tannenbaum S. R. (1982). Analysis of nitrate, nitrite, and [15N] nitrate in biological fluids. *Analytical Biochemistry*.

[29] Mauel J. (1990). Macrophage-parasite interactions in Leishmania infections. *Journal of Leukocyte Biology*.

[30] Leite A. L. J., Oliveira D. S., Mota L. R. W. (2020). Ectonucleotidases from trypomastigotes from different sources and various genetic backgrounds of _Trypanosoma cruzi_ potentiate their infectivity and host inflammation. *Cytokine*.

[31] Nikolova M., Carriere M., Jenabian M. A. (2011). CD39/adenosine pathway is involved in AIDS progression. *PLoS Pathogens*.

[32] Russo-Abrahao T., Cosentino-Gomes D., Gomes M. T. (2011). Biochemical properties of Candida parapsilosis ecto-5'-nucleotidase and the possible role of adenosine in macrophage interaction. *FEMS Microbiology Letters*.

[33] Thammavongsa V., Kern J. W., Missiakas D. M., Schneewind O. (2009). Staphylococcus aureus synthesizes adenosine to escape host immune responses. *The Journal of Experimental Medicine*.

[34] Reutershan J., Vollmer I., Stark S., Wagner R., Ngamsri K. C., Eltzschig H. K. (2009). Adenosine and inflammation: CD39 and CD73 are critical mediators in LPS-induced PMN trafficking into the lungs. *The FASEB Journal*.

[35] Antonioli L., Blandizzi C., Pacher P., Hasko G. (2013). Immunity, inflammation and cancer: a leading role for adenosine. *Rev. Cancer*.

[36] Barletta K. E., Ley K., Mehrad B. (2012). Regulation of neutrophil function by adenosine. *Arteriosclerosis, Thrombosis, and Vascular Biology*.

[37] Mizumoto N., Kumamoto T., Robson S. C. (2002). CD39 is the dominant Langerhans cell-associated ecto-NTPDase: modulatory roles in inflammation and immune responsiveness. *Nature Medicine*.

[38] de Figueiredo A. B., Souza-Testasicca M. C., Afonso L. C. C. (2016). Purinergic signaling and infection by _Leishmania_ : A new approach to evasion of the immune response. *Biomed J.*.

[39] Levesque S. A., Kukulski F., Enjyoji K., Robson S. C., Sevigny J. (2010). NTPDase1 governs P2X7-dependent functions in murine macrophages. *European Journal of Immunology*.

[40] Eichin D., Laurila J. P., Jalkanen S., Salmi M. (2015). CD73 activity is dispensable for the polarization of M2 macrophages. *PLoS One*.

[41] Murphy P., Wang J., Bhagwat S. (2017). CD73 regulates anti-inflammatory signaling between apoptotic cells and endotoxin-conditioned tissue macrophages. *Cell Death and Differentiation*.

[42] Chekeni F. B., Elliott M. R., Sandilos J. K. (2010). Pannexin 1 channels mediate 'find-me' signal release and membrane permeability during apoptosis. *Nature*.

[43] Qu Y., Misaghi S., Newton K. (2011). Pannexin-1 is required for ATP release during apoptosis but not for inflammasome activation. *Journal of Immunology*.

[44] Basu M., Gupta P., Dutta A., Jana K., Ukil A. (2020). Increased host ATP efflux and its conversion to extracellular adenosine is crucial for establishingLeishmaniainfection. *Journal of Cell Science*.

[45] Marques-da-Silva C., Chaves M. M., Chaves S. P. (2011). Infection with Leishmania amazonensis upregulates purinergic receptor expression and induces host-cell susceptibility to UTP-mediated apoptosis. *Cellular Microbiology*.

[46] Chow S. C., Kass G. E., Orrenius S. (1997). Purines and their roles in apoptosis. *Neuropharmacology*.

[47] Chaves S. P., Torres-Santos E. C., Marques C. (2009). Modulation of P2X (7) purinergic receptor in macrophages by Leishmania amazonensis and its role in parasite elimination. *Microbes and Infection*.

[48] Zheng L. M., Zychlinsky A., Liu C. C., Ojcius D. M., Young J. D. (1991). Extracellular ATP as a trigger for apoptosis or programmed cell death. *The Journal of Cell Biology*.

[49] Gomes R. S., de Carvalho L. C., de Souza V. R., Fietto J. L., Afonso L. C. (2015). E-NTPDase (ecto-nucleoside triphosphate diphosphohydrolase) of Leishmania amazonensis inhibits macrophage activation. *Microbes and Infection*.

